# Deformation Behavior of Human Dentin under Uniaxial Compression

**DOI:** 10.1155/2012/854539

**Published:** 2012-01-24

**Authors:** Dmitry Zaytsev, Sergey Grigoriev, Peter Panfilov

**Affiliations:** ^1^Ural State University, Lenin Avenue 51, Ekaterinburg 620000, Russia; ^2^Ural State Medical Academy, Repin Street 3, Ekaterinburg, 620219, Russia

## Abstract

Deformation behavior of a human dentin under compression including size and rate effects is studied. No difference between mechanical properties of crown and root dentin is found. It is mechanically isotropic high elastic and strong hard tissue, which demonstrates considerable plasticity and ability to suppress a crack growth. Mechanical properties of dentin depend on a shape of samples and a deformation rate.

## 1. Introduction

As a rule, human teeth are subjected by compressive load and friction during mastication of a food, whereas dentin, which is the hard base of a tooth, works under compression stress only [1]. Usually, compression stress into a tooth is limited by 30 MPa, but sometimes stress can reach the level allowed ever cutting off an annealing steel wire [2]. Therefore, uniaxial compression is the more suitable deformation scheme for *in vitro* study of the mechanical properties of dentin.

Mechanical behavior of human dentin is the subject for much researches in materials science and mechanics. Experiments have shown that it is the strong hard tissue (ultimate compressive strength is 250–350 MPa) demonstrated insufficient elastic deformation prior the failure (<2%) [3, 4]. However, under indentation, dentin is able to recover its initial size near indent when indenter has been unloaded [5]. Dentin samples can also exhibit some flow behaviors, when they are compressed under constant load [3]. Under compression dentin samples are also elastic and some plastic [6]. Fracture mechanism of dentin as follows: the main crack grows by joining with the satellite cracks [7]. It gives the basis for suggestion that dentin can possess considerable plasticity. Indeed the similar fracture mechanism acts in the plastic metals such as silver, nickel, and gold [8, 9]. However, an ability of dentin of both considerable elastic deformation and plastic deformation was not revealed in compression experiments. It is well known that mechanical properties of materials depend on the geometry (shape and size) of sample and the rate of loading, but none these effects were observed in dentin.

The aim of this work is the study of the deformation behavior of human dentin under compression including shape, size, and rate effects.

## 2. Materials and Methods

**Figure 1:**
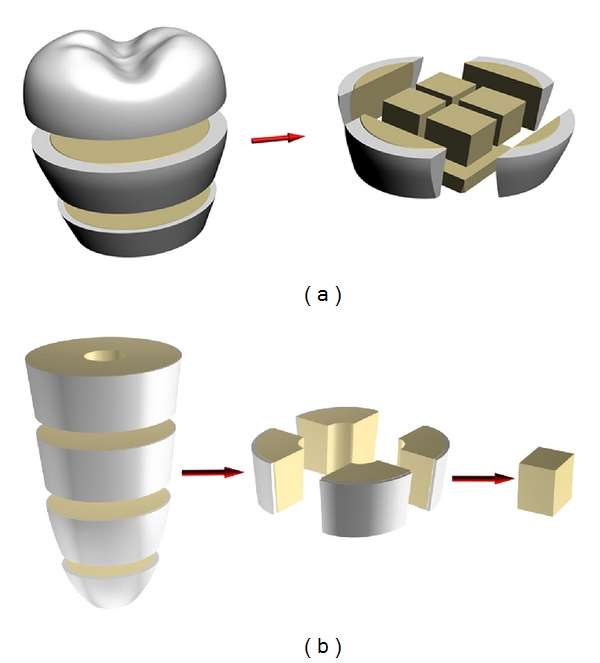


**Figure 2:**
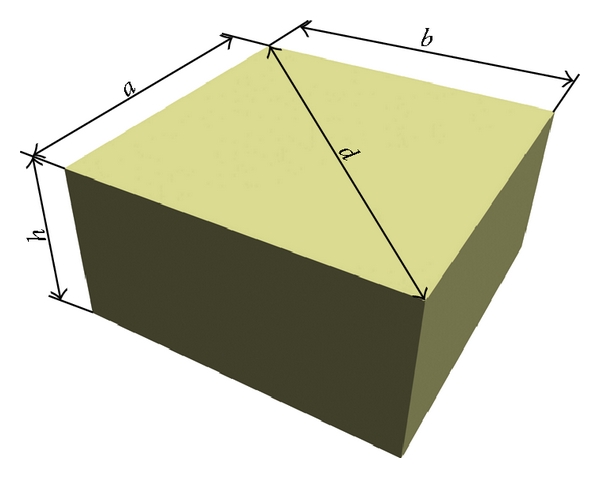


**Figure 3:**
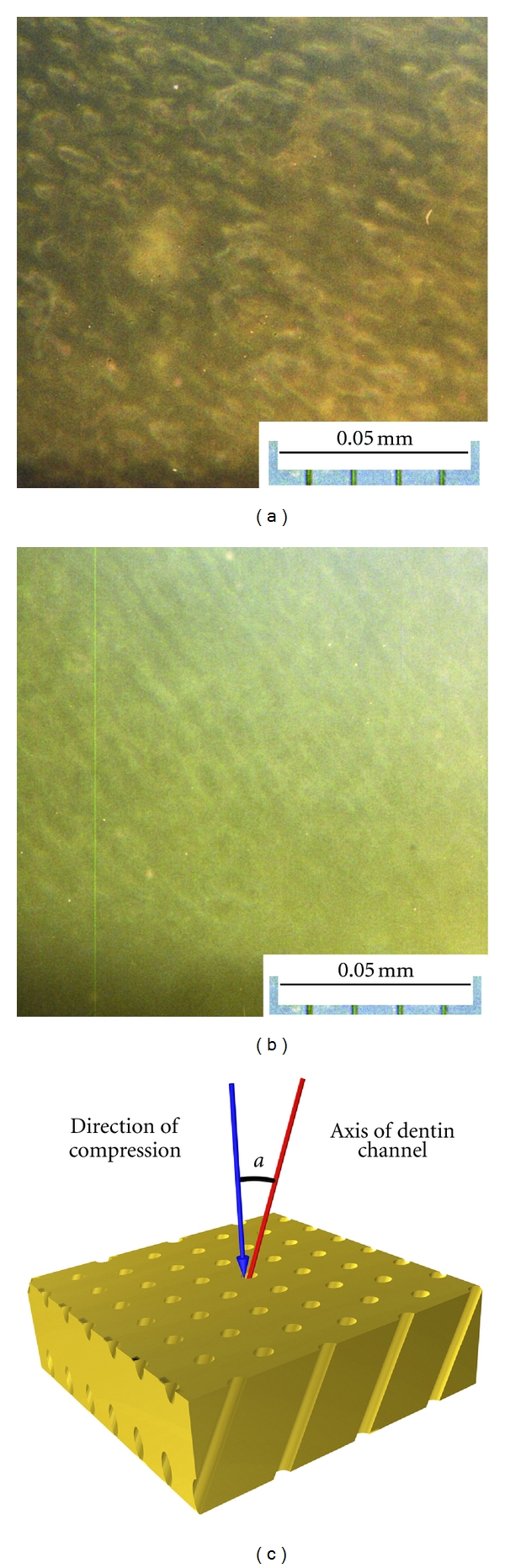


Five dozen teeth, which has been extracted according to the medical diagnosis and ethic protocol of USMA from the patients (both male and female in equal proportion) aged 20–40 years, were used in this research protocols. Samples for mechanical tests with the shape close to a cuboids were cut by a diamond saw from the crowns and roots of the freshly extracted intact (caries free) human teeth according to the schemes given in Figures 1(a) and 1(b), respectively. They were grouped in sets. The samples of the main set had the size of 2 × 2 × 0.65 mm^3^ and ratio *d*/*h* = 4.3 (see Figure 2). Such choice of the sample geometry is connected with the fact that almost 16 samples can be cut from a premolar. Samples with other sizes and ratios *d*/*h* were prepared for examination of the size and shape effects. Prior to the test, surfaces of the samples were abraded by abrasive paper with the grain size of 10 *μ*m for removing the damaged layer, which appeared after cutoff. The angle between the compression axis and the direction of dentin channels in the sample was determined by metallographic means (Figure 3). It could vary from zero (compression axis coincides with the dentin channel direction) to 90°. Shimadzu AGX-50 kN testing machine has been used for the room temperature (~25°C) mechanical tests (compression precision: maximum ±1%  of indicated value). Twenty samples taken from the main set were compressed under the traverse rate 0.1 mm/min. The set of samples with constant *d*/*h* ratio, but different sizes and the set of samples having different *d*/*h* ratio were tested at the same traverse rate (0.1 mm/min). Some samples from the main set were compressed at different traverse rates varied from constant load (5 hours under 200–450 MPa) to 10 mm/min with the step of one order. Surfaces of the samples were examined on light microscopes prior to and after each test. The size of the samples was controlled with a help of the instrumental microscope UIM-21 (precision of measurement ~1 *μ*m), too.

## 3. Results

**Figure 4:**
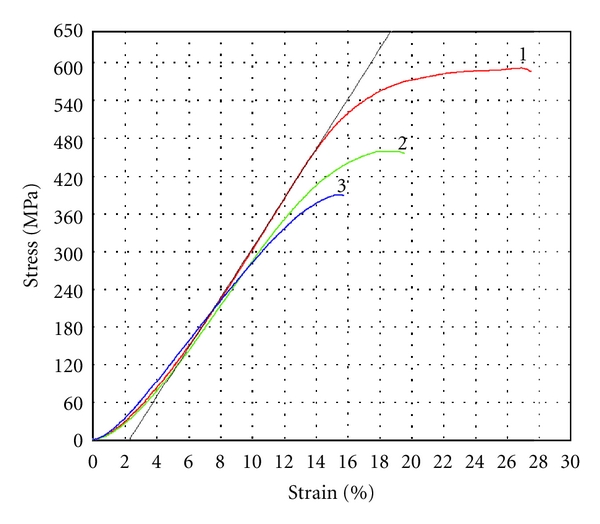


**Figure 5:**
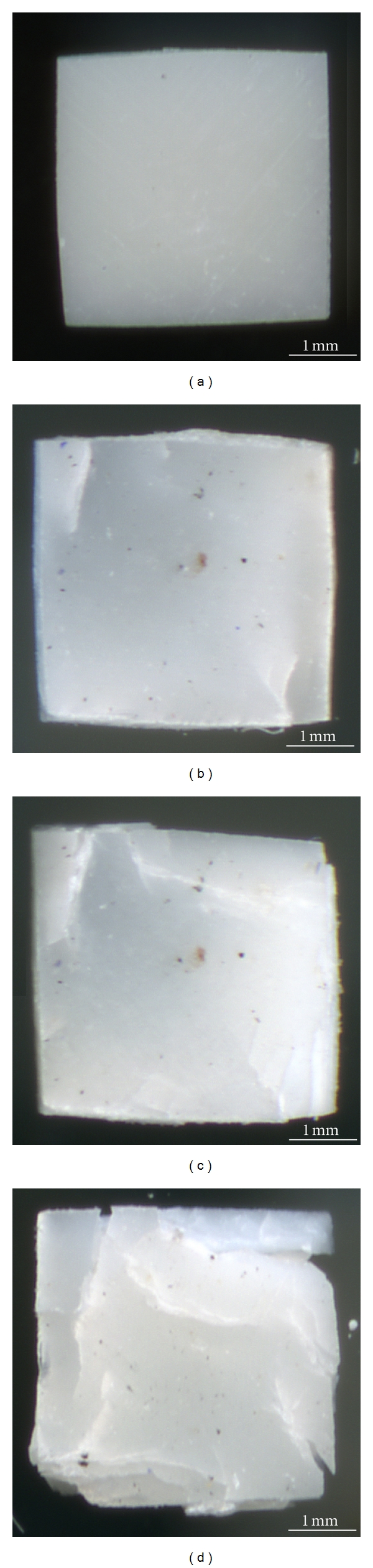


Compression tests of twenty samples taken from the main set have been carried out. Test was stopped as soon as the jog (a drop of stress) appeared on the deformation curve. No any differences between the curves for the crown and root dentin were obtained. The relationship between orientation of dentin channels in the sample and mechanical behavior of dentin was not detected. Therefore, mechanical properties of human dentin under compression could be characterized by the typical deformation curve, which belongs one of the samples from the main set, is given in Figure 4 (curve 1). Metallographic examination of the samples prior to and after the test has shown that there are cracks on the surfaces of compressed samples (Figures 5(a) and 5(b)). Hence, nucleation and growth of cracks may be considered as the main cause of jog on the deformation curve. Despite the appearance of cracks, dentin samples do not separate under compression.

Three regions are observed on the deformation curve. The trend of the curve in the first region (from zero to 3–5% at 50–100 MPa) is nonlinear. Such behavior can be caused by unperfected shape of the small sized samples. For example, they were not planoparallel to a sufficient degree to obtain homogenous distribution of stresses under loading. Nonlinear behavior of the deformation curve at the first region should be considered as “technological” factor, which does not reflect the inherent property of dentin. Indeed, the shape of the curve in the second region can be approximated by the straight line. The longitude of this region is from 3–5% to 10–13% at 50–100 MPa and 350–400 MPa. Young's modulus (4,02 ± 0,24 GPa) and proportional limit (386 ± 21 MPa at  *ε*  =11,6 ± 1,0%) have been calculated for this region. The third region is nonlinear (its longitude is from 10–13% to 25–30% at from 350–400 MPa to 550–600 MPa). The point of the high limit of stress for the third region (before the jog) has been accepted as the ultimate compressive strength of dentin (582 ± 27 MPa at *ε* = 27,5 ± 2,2%). The measurements of the thickness of samples prior to and after tests have shown that their irreversible deformation is 13,2 ± 1,9% (the elastic limit 442 ± 33 MPa) and, hence, elastic contribution into deformation behavior of the dentin samples is 14,2 ± 1,0%. Therefore, it may be concluded that the first and the second regions are the stage of elastic deformation (longitude 11,6 ± 1,0%), whereas the third one is the stage of elastic and plastic (viscoelastic) deformation (longitude 15,9 ± 2,5%).

**Figure 6:**
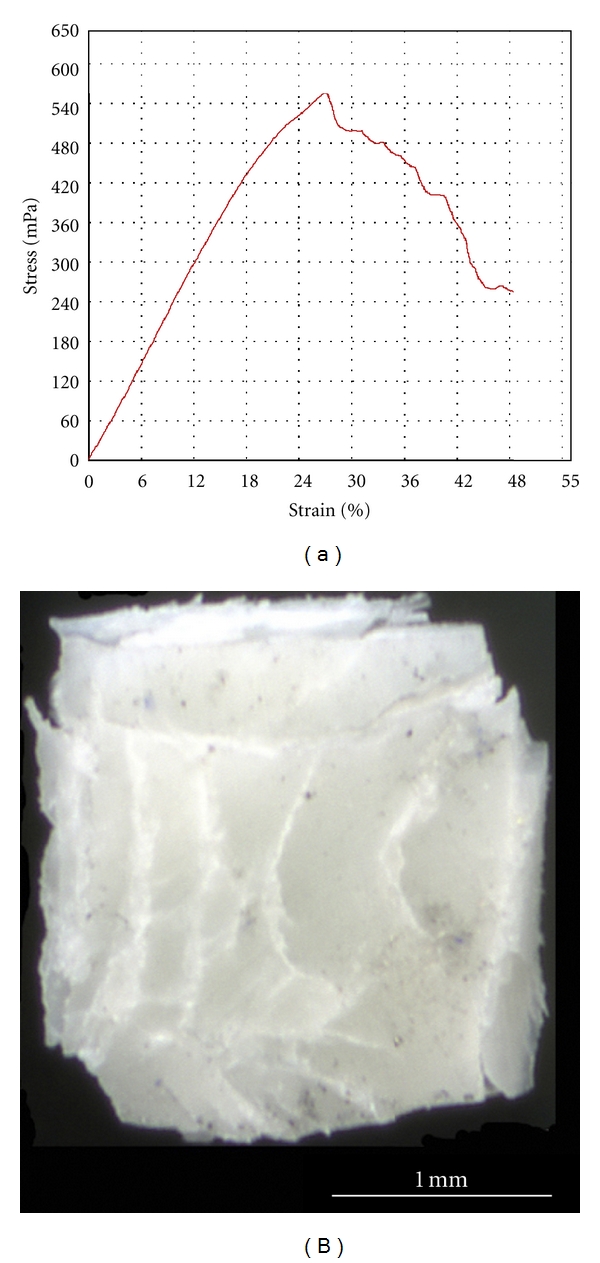


Compression test of the deformed sample can be carried out once more, insomuch as it did not fail after the first compression. The second test was stopped as soon as the jog on the deformation curve appeared. The samples also do not separate under loading, while the quantity and length of cracks on their surfaces increase in comparison with the first test (see Figure 5(c)). The shapes of the typical deformation curves for the second and the first compressions are similar (see Figure 4, curve 2). The trend of the deformation curve for the second test is close to the first test on the elastic stages, whereas its trend for the viscoelastic stage is different. The maximal stress (438 ± 22 MPa) and full strain (19,0 ± 0,8%) for the second compression are lower than for the curve 1. The third compression of this sample obeys the tendency described above (see Figure 4, curve 3). No failures of the samples were observed after the third compression, but the quantity and length of cracks on their surfaces increased (see Figure 5(d)). Some mechanical properties of the human dentin obtained during three compressions of the samples from the main set are grouped in Table 1. It is visible that the inclination of the linear part of deformation curve (Young's modulus), the proportional limit, and the compressive strength decrease after every test. The elasticity of dentin continues to be the same, while the drop of plasticity of dentin took place after the first compression. Therefore, the total deformation of the dentin samples under compression drops after the first test and continues decreasing after the following tests. Some samples from the main set were compressed up to the moment when their separation begins at strain > 50% (see Figure 6(a)). Inspite of, this lot of stable cracks can exist in the samples (see Figure 6(b)).

**Figure 7:**
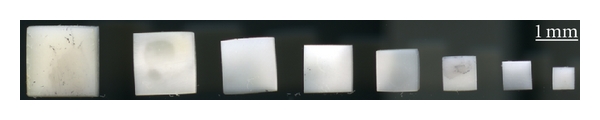


**Figure 8:**
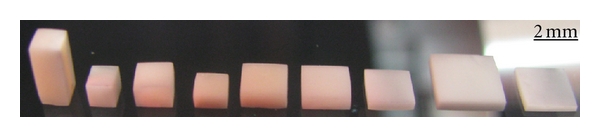


**Figure 9:**
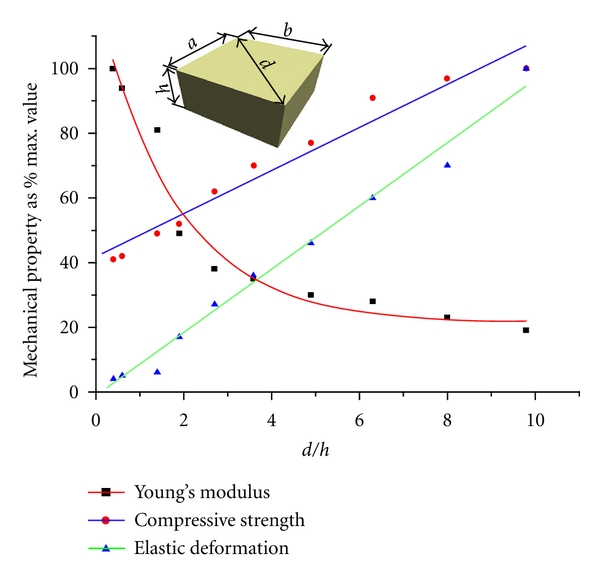


The size effect under compression has been studied. Mechanical properties of the dentin samples having the ratio *d*/*h* = 3.5 and the size of grains varies from 4 to 1 mm (see Figure 7), is given in Table 2. Analysis of the findings allows concluding that the deformation behavior of dentin does not depend on the size of sample, when *d*/*h* ratio is constant. The samples having ratios *d*/*h* from 0.4 to 10 are shown in Figure 8, while their mechanical properties are documented in Table 3. It is clearly visible, that the maximal values of the total (elastic and plastic) deformation and the compressive strength, but the minimal value of the Young's modulus have been reached in the samples with *d*/*h* = 9.8. The character of dependences “compressive strength ratio *d*/*h*” and “deformation ratio *d*/*h*” can be approximated by a straight line, whereas dependence “Young's modulus ratio *d*/*h*” is close to the exponential curve (see Figure 9). Hence, no size effect is observed, while the shape effect displays in human dentin under compression.

**Figure 10:**
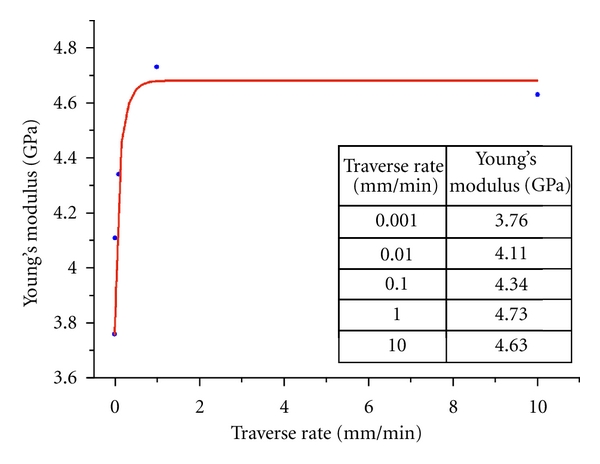


**Figure 11:**
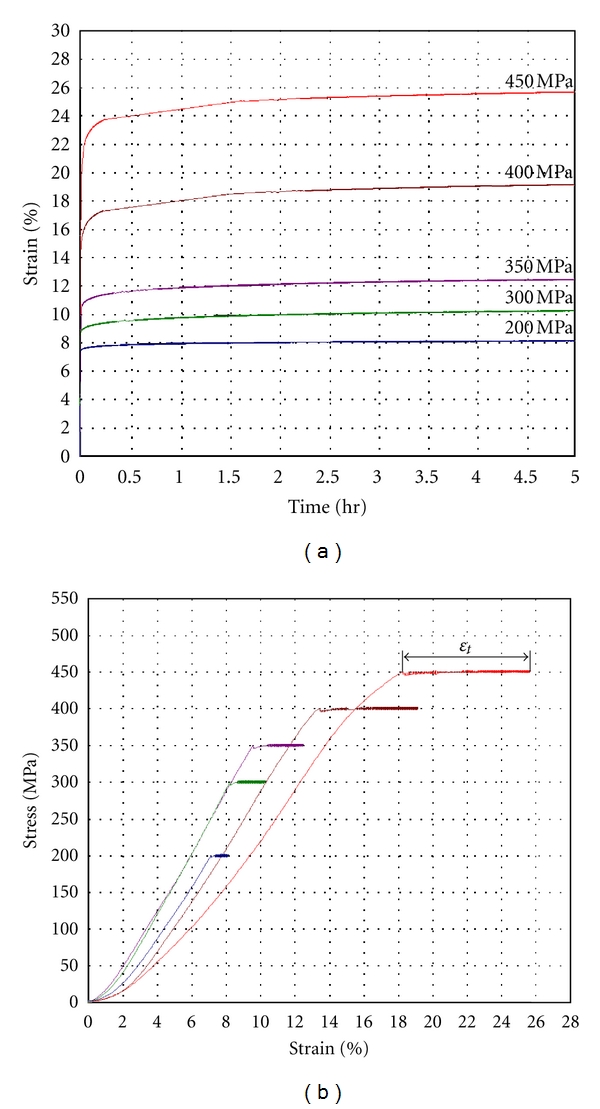


**Figure 12:**
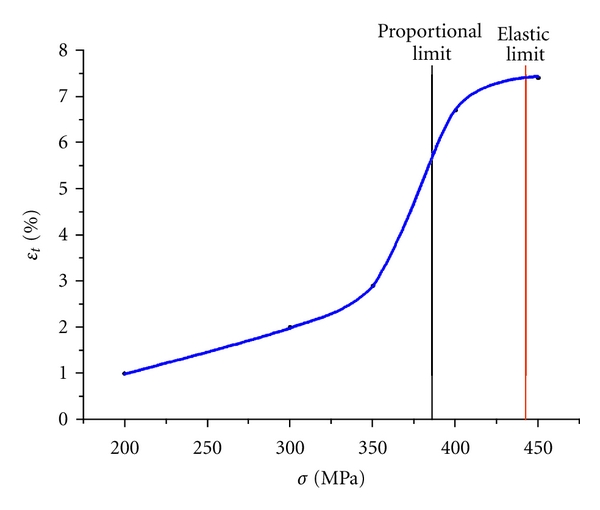


Compression tests of the samples from the main set (2 × 2 × 0.65 mm^3^, *d*/*h* = 4.3) under different traverse rates have been carried out. It was shown that this parameter influences the Young's modulus only, while the compression strength and the total deformation, including elastic and plastic contributions, do not depend on the traverse rate. Young's modulus is increased from 3.8 GPa at 0.001 mm/min to 4.7 GPa at 1 mm/min and further it does not depend on the traverse rate (Figure 10). The same dentin samples can hold the applied stress without failure under the creep tests (Figure 11(a)). The curves have shown the characteristic pattern for viscoelastic materials: strain increasing with time until the stress is held constant. Metallographic examination of their back surfaces after testing has shown that cracks are absent here. Indeed, the maximal applied load (450 MPa) is less than the ultimate compressive strength of the samples from the main set, when the cracks should appear in dentin. However, the sample begins to divide when applied stress reaches the ultimate compressive strength. The magnitude of deformation under constant loading (*ε*_*t*_) depends on the applied stress and varied from 1% at 200 MPa to 8% at 450 MPa (see Figure 11(b)). Dependence of the viscous compliance under constant loading from applied constant load is given in Figure 12. The strain is inconsiderable at loads of 200–300 MPa (≤2%). It begins growing near the proportional limit and reaches 8% at the elastic limit.

## 4. Discussion

The findings on deformation behavior of both root and crown dentin under compression have shown that human dentin is the high elastic and, simultaneously, plastic strong hard tissue, where the crack growth has been effectively suppressed. The similar behavior, when high elasticity coexists together with considerable plasticity, is inherent to some filled polymers, although these materials never exhibit so high strength as dentin [10]. The shape and the rate effects should be taken into account for modeling of mechanical properties of human dentin as it takes place for polymers [11]. No anisotropy of mechanical properties was detected in our experiments. However, it does not mean that human dentin, which is composite having hierarchic structure, should be considered as isotropic tissue on all scales. The mechanism, which could explain such deformation behavior of dentin, needs a detailed study of its microstructure with a help of X-rays analysis and transmission electron microscopy.

Some obtained results can be considered as the contradiction with the data on mechanical properties of human dentin under compression [12]. However, examination of well-known papers on deformation of dentin does not maintain this point of view. Indeed, the Peyton's work described the samples with the ratio *d*/*h* = 1.00–0.25, where the total deformation prior to the failure was <1%, Young's modulus 12–16 GPa and compression strength ~300 MPa [3, 13]. Presented findings are close to the mentioned-above ones (see Table 3). Another reference, where dentin samples had the ratio *d*/*h*~3.00, reported the similar to Peyton's values for Young's modulus and the compressive strength after small total deformation ~3%, which does not coincide with the data in Table 3 [14]. However, the image of the sample after compression test (see Figure 5 in [14]) is demonstrated that it has divided under the test, whereas our samples having the same *d*/*h* ratio never failed under compression. Perhaps, the difference is caused by the procedure of sample preparation used in [14]. Indeed, the crack growth mechanism of dentin includes an ability of the tissue to brake the crack growth due to deformation processes and, hence, the arrest of cracks [15, 16]. On the other hand, the rate dependence of Young's modulus of human dentin is close to the viscoelastic materials [17]. In addition, presented findings agree with the deformation behavior of human dentin under indentation (point loading), according to which it is the high elastic and plastic hard tissue where crack growth has been suppressed [5, 18].

No difference between mechanical properties of root and crown human dentin is revealed, in spite of the discussion in the literature [19]. It agrees with microstructure examination of the human dentin according to which the main morphological features (diameter of dentin tubulars and distance between neighboring ones) in root and crown of a tooth are similar [20]. It seems to be that the difference between mechanical properties of root and crown human dentin is sooner caused by the shape and the preparation technique of samples than other reasons. Dependence of the elastic constants of dentin from orientation of the tubulars in the samples, which was revealed by means of the resonant ultrasound spectroscopy, is also discussed in the literature [21]. However, no any anisotropy of mechanical properties of human dentin under compression is observed in the current work and some other sources ([13], Stanford et al. 1960). It means that under uniaxial compression human dentin may be considered as mechanically isotropic hard tissue in spite of its hierarchic microstructure.

Living experience and clinical practice have shown that a human never uses the strength resource of dentin on 100%. Stress level in a tooth under mastication of some special food is no more than 100 MPa ever, when maximal strain is 5% and reversible. Therefore, degradation (failure) of human teeth under mechanical stresses is the aftermath of a dental pathology only.

## 5. Conclusions

The findings allow concluding that human dentin under uniaxial compression behaves as mechanically isotropic high elastic and strength hard tissue, which demonstrates considerable plasticity and ability to suppress a crack growth. No difference between deformation behavior of crown and root dentin is observed. Mechanical properties of dentin depend on the shape of samples and deformation rate.

## Figures and Tables

**Table 1:** 

	Young's modulus, GPa	Proportional limit, MPa	Compressive strength, MPa	Elastic deformation, %	Plastic deformation, %	Linear elastic deformation, %	Total deformation, %
First compression	4,02 ± 0,24	386 ± 21	582 ± 27	14,2 ± 1, 0	13,2 ± 1,9	11,6 ± 1,0	27,5 ± 2,2
Second compression	3,21 ± 0,22	298 ± 21	438 ± 22	14,7 ± 1,0	4,3 ± 0,7	11,0 ± 0,6	19,0 ± 0,8
Third compression	3,04 ± 0,27	249 ± 18	365 ± 27	13,9 ± 1,2	2,4 ± 0,9	9,8 ± 0,6	16,3 ± 0,6

**Table 2:** 

Size, mm			Young's modulus, GPa	Proportional limit, MPa	Compressive strength, MPa	Elastic deformation, %	Plastic deformation, %	Total deformation, %
a	b	h						
3.87	3.77	1.52	3.90	317	500	12.5	9.5	22
3.31	3.31	1.37	3.68	294	511	16.1	9.9	26
2.97	2.90	1.16	4.52	297	485	13.1	7.9	21
2.59	2.57	1.04	4.20	340	454	10.8	9.2	20
2.25	2.26	0.91	4.77	355	523	14.2	8.8	23
1.83	1.87	0.74	5.23	371	511	10.7	8.3	19
1.53	1.55	0.61	4.81	387	541	15.6	8.4	24
1.17	1.19	0.47	5.88	304	502	15.2	6.8	22

**Table 3:** 

*d*/*h*	Young's modulus, GPa	Proportional limit, MPa	Compressive strength, MPa	Elastic deformation, %	Plastic deformation, %	Total deformation, %
9.8	2.14	397	815	39.9	16.1	56
8.0	2.65	459	792	28.1	17.9	46
6.3	3.13	357	740	23.9	14.1	38
4.9	3.43	354	626	18.2	13.8	32
3.6	3.95	347	569	14.3	11.7	26
2.7	4.31	361	510	10.7	11.3	22
1.9	5.59	326	425	6.7	4.3	11
1.4	9.09	282	396	2.3	3.7	6
0.6	10.65	191	343	2.0	2.9	4.9
0.4	11.28	157	331	1.5	1.9	3.4
